# Oral Squamous Cell Carcinoma (OSCC) Imitates Denosumab-Induced Osteonecrosis of the Mandibular Alveolus: A Diagnostic Challenge

**DOI:** 10.7759/cureus.42619

**Published:** 2023-07-28

**Authors:** Vasileios Zisis, Dimitrios Andreadis, Anastasios Iliadis, Christos Angelopoulos, Athanasios Poulopoulos

**Affiliations:** 1 Oral Medicine/Pathology, Aristotle University of Thessaloniki, Thessaloniki, GRC; 2 Oral and Maxillofacial Radiology, National and Kapodistrian University of Athens, Athens, GRC

**Keywords:** oral cancers, denosumab and cancer, jaw osteonecrosis, medication-related osteonecrosis of the jaw, oral cavity squamous cell carcinoma

## Abstract

Oral squamous cell carcinoma (OSCC) may arise in the the alveolar ridge (in a minority of cases). Smoking, chronic mucosal injuries, and poor oral hygiene are involved in its pathogenesis. It mostly occurs to men instead of women and affects the mandible on a 3:2 ratio to the maxilla. The objective of the current study is to present an interesting case of an OSCC of the alveolar ridge mimicking jaw osteonecrosis due to denosumab, resulting in differential diagnostic dilemmas. A 78-year-old female patient, edentulous and bearing total dentures, was referred with a persistent (four months), severely painful, ulcerative lesion in the anterior lateral (right) region of the residual alveolar ridge of the mandible. Medical history referred to a long-term systemic steroid use due to sarcoidosis as well as the subcutaneous use of denosumab for osteoporosis one/month for one year. Cone-beam CT (CBCT) examination was performed where bone resorption was detected and a differential diagnosis of osteonecrosis of the jaws (ONJs) from denosumab or neoplasia was made. A biopsy was carried out, and the histological examination showed that soft tissues and underlying bone were infiltrated by abnormal, confluent, compact islands of malignant squamous cells with intense atypia and numerous mitoses indicating a moderately differentiated OSCC. Denosumab inhibits the binding of receptor activator of nuclear factor ligand (RANKL) to receptor activator of nuclear factor-kappa (RANK); this decreases bone resorption and results in increased bone density. However, denosumab may induce ONJ. The area of exposed bone and abnormal soft tissue alterations may resemble both benign and malignant diseases. Osteonecrosis may mimic OSCC or may even provide the suitable substrate for the development of OSCC. Biopsy as well as bone imaging examination are required to accurately determine the possibility of neoplastic formation and its boundaries in cases of osteonecrosis especially in patients under treatment with denosumab or bisphosphonate-related ONJ (BRONJ).

## Introduction

Oral cancer (OC) is a malignant process of the oral cavity (from the lips to the anterior pillars of the fauces) that disproportionately affects men [1,2]. It is the eighth most common cancer (>300,000 cases per year), characterized by a high mortality rate and detrimental effects on the patients' aesthetics [1-3]. Approximately 90-95% of the cancer cases, located in the oral cavity, are oral squamous cell carcinomas (OSCCs), which are characterized as well, moderately and poorly differentiated, based on their histopathological characteristics [4,5]. Curative resection and reconstruction continue to be the most common means of preserving the form and function of the head and neck area [1]. Due to local aggressiveness and metastasis, OSCC has a poor prognosis despite recent advances in treatment modalities; recurrence occurs in 30% of cases [6]. Local and regional recurrences are the leading cause of OSCC-associated mortality, with five-year survival dropping from 92% in patients without recurrences to 30% in patients with recurrences [1-6]. Osteonecrosis of the jaw (ONJ) is a rare condition observed in patients receiving osteoclast-targeted antiresorptive treatment [7]. The incidence of ONJ among patients taking oral antiresorptives such as Denosumab or bisphosphonates (bisphosphonate-related ONJ (BRONJ)) for the treatment of osteoporosis is low (approximately 1:100,000), whereas it is significantly higher (10%) among patients taking intravenous bisphosphonates for the treatment of metastatic bone diseases [7]. Denosumab, a human monoclonal antibody against receptor activator of nuclear factor ligand (RANKL), is employed as an antiresorptive agent [7,8]. Denosumab inhibits the binding of RANKL to receptor activator of nuclear factor-kappa (RANK); this decreases bone resorption and results in increased bone density [7,8]. However, denosumab, similarly to bisphosphonates, may induce ONJ [7-9]. The combination of denosumab with risk factors such as tooth extraction, poor oral hygiene, the use of removable devices, and chemotherapy may promote the development of ONJ [10]. When an area of exposed bone and abnormal soft tissue alterations are presented, it can be difficult to differentiate between malignant or non-malignant disease, and even histopathological comparison can be challenging [11]. There is even a report of a case where a case of medication-related ONJ (MRONJ) was misdiagnosed as OSCC, since the malignant-appearing lesion turned out to be benign [12]. The prevalence and incidence of such lesions are expected to rise as the use of antiresorptives and bisphosphonates in the community increases due to the prolonged life expectancy [11]. This study describes a case of OSCC of the mandible in a 78-year-old female who was treated with denosumab for the management of osteoporosis.

## Case presentation

**Figure 1:**
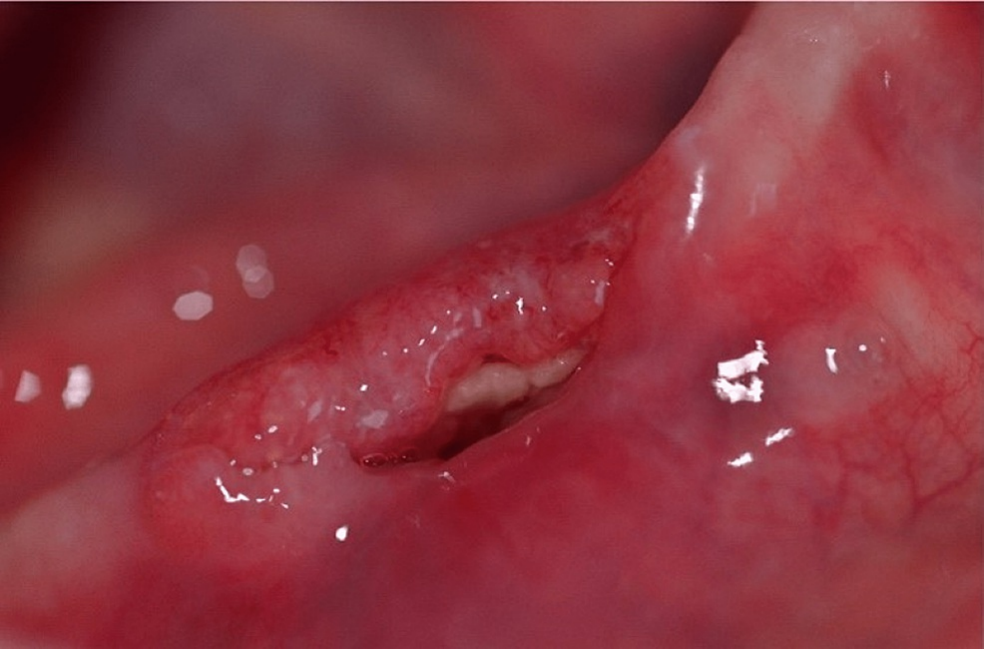
Ulcerative mucosal lesion with underlying osteonecrosis

A female patient, 78 years old, was referred, to the Department of Oral Medicine/Pathology, School of Dentistry, Aristotle University of Thessaloniki, Thessaloniki, Greece, complaining about severe pain in the right half of the mandible. The pain was described by the patient as constant, without any mention to specific alleviating or aggravating factors. Before the examination, the patient provided written informed consent. This form was approved by the School of Dentistry, Aristotle University of Thessaloniki and was in accordance with the Helsinki Declaration for research and patient’s ethics. Subsequently, the patient was examined thoroughly. Her medical history revealed a long-term systemic steroid use (methylprednisolone) due to sarcoidosis as well as the subcutaneous use of denosumab for osteoporosis 60 mg once a month for one year. The patient was edentulous bearing total dentures. A persistent (four months), severely painful, ulcerative lesion was noticed in the anterior lateral (right) region of the residual alveolar ridge of the mandible, without cervical lymphadenopathy (Figure 1).

**Figure 2:**
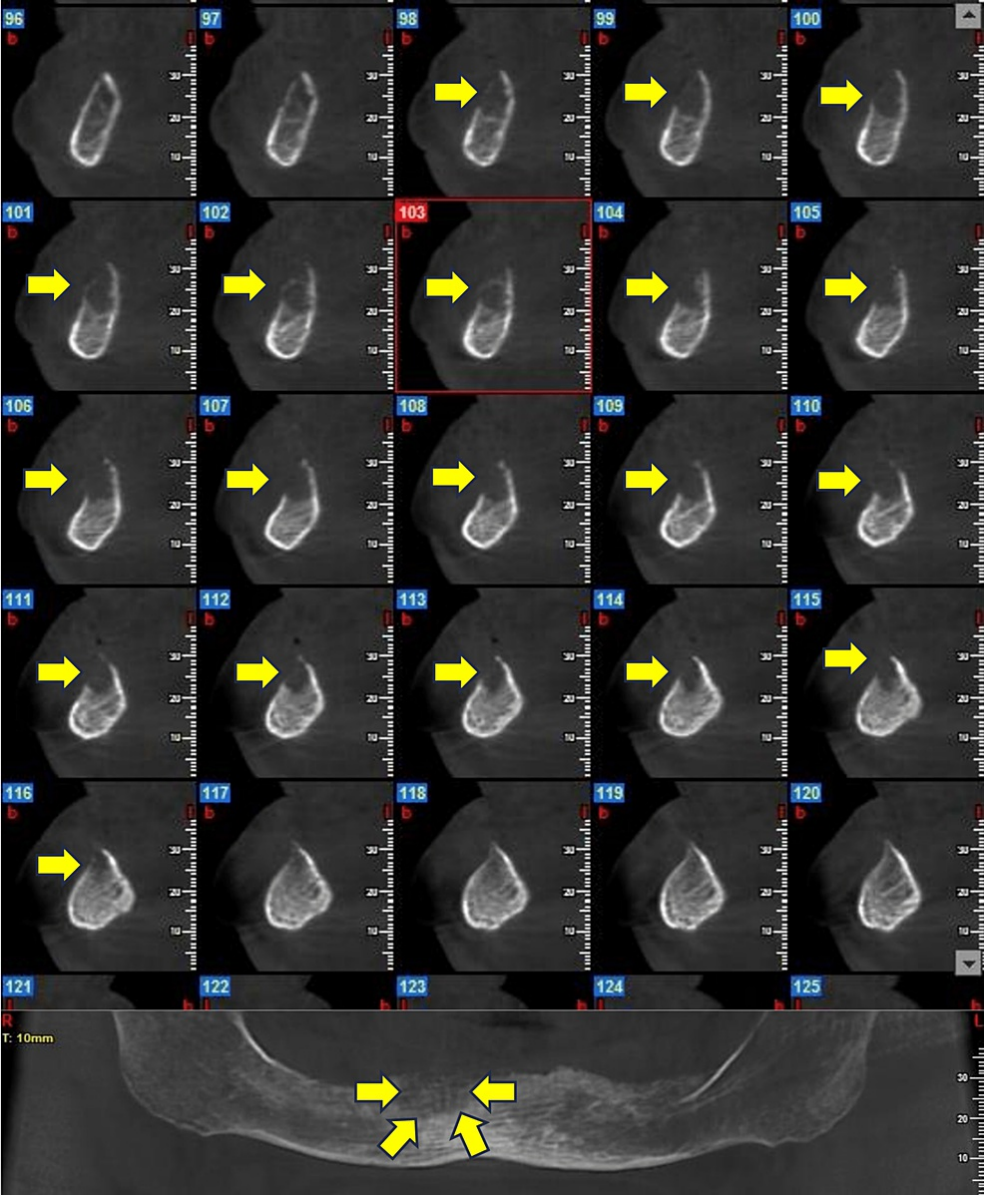
Three-dimensional radiographical imaging of the osteolytic lesion in the fourth quadrant (yellow arrows indicate the radiolucent lesion)

Cone-beam CT (CBCT) examination was performed where bone resorption was detected and a differential diagnosis was hypothesized among ONJ from denosumab, trauma and malignancy (Figure 2).

**Figure 3:**
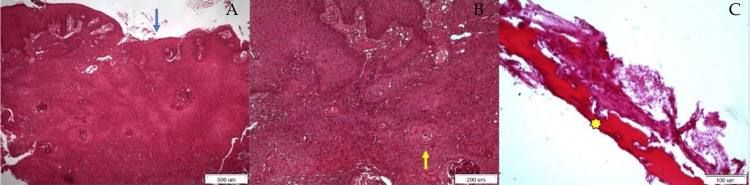
Histological examination showing ulceration (Figure 3A, blue arrow), neoplastic cell islands manifesting severe atypia and numerous mitotic divisions invading gingival mucosa (Figure 3B, yellow arrow) and alveolar ridge of mandible (Figure 3C, yellow asterisk)

A biopsy was carried out and the histological examination showed an ulcerative lesion (Figure 3A), with infiltration of gingival mucosa (Figure 3B) and of the residual alveolar ridge of the right half of the mandible (Figure 3C) by abnormal islands of neoplastic cells with intense atypia and many mitoses settling the diagnosis of a moderately differentiated case of OSCC.

## Discussion

The possibility of osteonecrosis ranges from 0.01% in osteoporosis patients to 15% in cancer patients receiving bisphosphonates or antiresorptives such as denosumab [13]. Additional risk factors include dental surgery, odontogenic infection, and concurrent chemotherapy or corticosteroid administration. Immunosuppression could be a risk factor for OSCC development [14,15]. In most cases, MRONJ is asymptomatic, but it may also manifest severe pain, paresthesia, spontaneous tooth loss, and pathological fracture [13]. OSCC involving the mandibular alveolar process may manifest similar clinical signs [15]. Therefore, all osteolytic lesions in patients receiving bisphosphonates or antiresorptives must be examined histopathologically [11]. On the other hand, malignancy suspicion should always be high, regardless of whether the lesion is a primary OSCC or a metastatic osseous lesion [11]. The possibility of OSCC arising in a pre-existing MRONJ has been also reported [16]. Denosumab is associated with numerous adverse effects, such as ONJ. Although the literature indicates that the incidence of ONJ due to denosumab use is low, when it does occur, it is associated with severe functional and masticatory disorders that have a significant impact on patient quality of life [10]. For this reason, it is essential to establish a set of criteria to identify at-risk patients, thereby reducing the incidence of such cases, as well as protocols for early treatment. It has been reported that most of the adverse effects of denosumab are observed at doses of 120 mg whereas the duration of treatment is the key factor with regards to 60 mg doses, since the duration is directly proportional to the incidence of such adverse effects [10]. Denosumab is regularly prescribed to cancer patients, for the purpose of delaying or preventing skeletal-related events, such as bone metastases, pathologic bone fracture, spinal cord compression, surgical and radiologic treatment for bone lesions, as well as hypercalcemia [10]. However, the question whether denosumab may delay the progress of the locoregional osteolysis induced by a OSCC of the mandible mucosa as in our case cannot be clarified.

## Conclusions

OSCC is the prevalent and most widespread malignancy in the oral cavity and can affect the jaws. On the other hand, the presence of MRONJ is increased due to the wide use of relevant medications against osteoporosis and metastases. Denosumab, combined with risk factors such as the use of removable prosthetic devices and adjuvant radio/chemotherapy, may induce ONJ. On the other hand, the question arises, of the possible role of denosumab in slowing down the progression of osseous invasion in case of OSCC, hence winning some time for the patient and rendering the medically compromised situation more manageable. The individual medical history may always be taken into consideration (i.e., smoking) but should also not blind the clinician with regards to the probabilities underlying the differential diagnosis. A combined approach including preventive measures for the elimination or reduction of the risk factors, clinical screening, biopsy and imaging as well as the harmonious cooperation of different dental and medical specialties shall provide the patient with the most favorable prognosis and the least repercussions.
